# Marker of vitamin D status in healthy children: Free or total 25-hydroxyvitamin D?

**DOI:** 10.1371/journal.pone.0202237

**Published:** 2018-08-23

**Authors:** Laura Mantecón, Mª Agustina Alonso, Vanessa Moya, Ana Gloria Andrés, Noelia Avello, Eduardo Martínez-Morillo, Fernando Santos

**Affiliations:** 1 Department of Pediatrics, Hospital Universitario Central de Asturias (HUCA), Health Service of the Principality of Asturias, SESPA, Oviedo, Spain; 2 Department of Pediatrics Complejo Asistencial Universitario León, (CAULE), Health Service of Castilla-León, SACYL, León, Spain; 3 Department of Biochemistry, Hospital Universitario Central de Asturias, Health Service of the Principality of Asturias, Oviedo, Spain; Charles P. Darby Children's Research Institute, UNITED STATES

## Abstract

**Objective:**

To assess if serum free 25-hydroxyvitamin D (25OHD) is a better indicator of vitamin D status than total 25OHD in healthy children.

**Methods:**

Cross-sectional prospective clinical study was designed. We measured serum free 25OHD concentrations and its correlation with calculated free 25OHD, total 25OHD, intact parathyroid hormone (PTH), and vitamin D binding protein (DBP) in children. The influence of age, sex, ethnicity, body mass index (BMI), season of the year, diet intake, vitamin supplements, time spent outdoors and albumin concentrations on free 25OHD was also analyzed. 241 children aged from 0 days to 14 years, and living in the northern Spain (latitude 43° N), were included.

**Results:**

Mean (SD) free 25OHD concentrations were 2.48 (1.39), 5.46 (3.12), 4.12 (1,72), 3.82 (1.43) pg/ml in children aged 0 days, 1 month-2 years, 2–6 years and >6 years, respectively. Correlation between directly measured and calculated free 25OHD was high and significant (r = 0.66) as well as the correlation between serum free and total 25OHD concentrations (r = 0.61). No significant correlation was found between PTH and free 25OHD (r = -0.08). The total 25OHD and PTH concentrations’ correlation was inverse (r = -0.25) and significant. Neither free nor total 25OHD concentrations correlated with DBP concentrations. Among the analyzed variables, free 25OHD values were higher in spring/summer than in autumn/winter in children older than 6 years.

**Conclusions:**

: These findings do not support that free 25OHD is a better marker of vitamin D deficiency than total 25OHD in healthy pediatric population.

## Introduction

The status of vitamin D is clinically estimated by measuring total serum 25-hydroxyvitamin D (25OHD). Vitamin D plays a key role in the regulation of mineral metabolism [1], but in recent years a growing number of clinical studies have claimed an association between low vitamin 25OHD concentrations and increased susceptibility to certain acute and chronic diseases [2,3,4]. This potential role of vitamin D has prompted an increasing interest in detecting vitamin D deficiency.

25OHD circulates in blood mostly bound (85–90%) to its specific binding protein (DBP) and weakly to plasma albumin (10–15%), while a small fraction (<1%) constitutes its “free form” [5–7]. The bioavailable fraction is the sum of the free 25OHD and that bound to albumin [8,9]. The “free-hormone hypothesis” states that the biologic activity of hormones lies in the free or non-bound fractions to DBP [10], as it occurs with sex steroids or thyroid hormones [11,12], DBP is a protein with various genetic variations or polymorphisms that appear to modify their affinity for 25OHD and thus its free fraction [13], This might explain that similar concentrations of bioavailable 25OHD have been found in two groups of white and black people, despite lower serum levels of total 25OHD and DBP in the group of black individuals, which also had higher bone mineral density [14]. Variations in DBP concentrations and/or its different genotypes might be confusing factors on the interpretation of serum total and free 25OHD concentrations [6]. In addition, the normal values of DBP concentrations vary with age [9].

This “free-hormone hypothesis” suggests that the binding to DBP impairs the delivery of 25OHD to vitamin D-activating 1-alpha-hydroxylase in target cells, and therefore the free vitamin D fraction would better reflect the functional status of vitamin D. Thus, the reliability of assessing vitamin D status by measuring total serum 25OHD levels is questioned [15]. Free 25OHD or its bioavailable fraction was more strongly associated with bone mineral density and mineral bone parameters than the total 25OHD levels in healthy adults [16], patients with kidney diseases [17] and postmenopausal women [8]. Recent studies have reported directly measured free vitamin D in adults [18–21] or sick children [22], but there are no studies on free 25OHD and DBP levels in healthy children so far.

## Objectives

The present study was designed to determine serum free 25OHD concentrations in a healthy paediatric population living in a poorly sunlit geographical area and to analyze the correlation between serum concentrations of free 25OHD and serum total 25OHD, PTH and DBP concentrations as well as the correlation between directly measured and mathematically calculated free 25OHD concentrations. Secondary outcomes were to assess the influence of variables such as sex, age, ethnicity, BMI, season of the year, sun exposure, vitamin D supplements, diet, and albumin concentrations on free 25OHD levels.

## Material and methods

### Design, setting and subjects

A clinical, prospective, and cross-sectional study approved by the Regional Ethics Committee of the Principality of Asturias was conducted. Written informed consents were obtained from parents. Two hundred and forty one healthy children (141 males) aged 0 to 14 years were enrolled in the study, during a period of 15 months, from December 2014 to March 2016. The study was carried out in two tertiary hospitals located in northern Spain (Asturias and Leon, latitude, 43° and 42°N, respectively).

Blood samples were obtained from umbilical cord blood at birth in newborns, whereas the samples from the rest of patients were collected during routine preoperative laboratory tests from a peripheral vein.

Infants and children chosen to participate in the study were healthy individuals who got their blood tested before programmed surgery for minor problems (adenoidectomy, inguinal hernia, hydrocele, etc…). Inclusion criteria were: born at term (newborns), the signing of informed consent from parents and residence in Asturias and Leon. Those children with conditions that might affect vitamin D or bone metabolism were excluded. Serum free and total 25OHD, PTH and DBP concentrations were measured as detailed below.

The following variables were collected from the mothers by telephone questionnaire survey: affiliation, sex, ethnicity, pregnancy-related diseases and vitamins or nutritional supplements during pregnancy in the group of newborns. Balanced diet intake, time spent outdoors and use of vitamin supplements were asked to the rest of the children’s parents. At the time of blood extraction, body weight and height were measured and body mass index (BMI) was calculated as weight/height^2^ (kg/m^2^). Overweight was defined as BMI 80th-97th percentile for age in boys and ≥85th percentile for age in girls. Obesity was defined as BMI ≥97th percentile for age and sex. BMI percentiles were determined by using age-gender-specific growth charts for the Spanish population [23].

### Laboratory measurements

Serum total 25OHD and PTH concentrations were measured by an electro-chemiluminescence assay (Roche Diagnostics Laboratory, Mannhein, Germany). For serum 25OHD, the inter-assay coefficient of variation (CV) determined with PreciControl Varia 1 and 2 ranged from 2.1 to 8.7%. For serum PTH, inter-assay CV determined with PreciControl Varia 1 and 2 ranged from 1.5 to 4%. Direct measurement of free 25OHD concentration was performed by a competitive ELISA assay (KARF1991, DiaSource, Louvain-la-Nueve, Belgium), which detects free fraction 25OH vitamin D. Intra-assay CV for controls ranged from 0.7 to 11.6%.

Levels of vitamin D binding protein (DBP) were measured by liquid chromatography (LC)-tandem mass spectrometry (MS/MS). Samples were subjected to a process of protein denaturation, trypsin digestion and purification. An isotopically labelled peptide common to the three DBP isoforms (GQELCADYSENTFTEYK) was added in order to perform relative quantification. Pretreated samples were analyzed on an expert ultra 100 (Exigent, ABSCIEX) coupled online to a triple-quadruple mass spectrometer (QTRAP 5500, ABSCIEX) using an electrospray ionization source and selected reaction monitoring (SRM) detection mode.

Mathematical calculation of free 25OHD was carried out using the following formula [24]:

$$Free\;vitamin\;D=\frac{total\;vitamin\;D}{1+(Ka_{\mathrm{alb}}{}^{*}\times \mathrm{albumin})+({\mathrm{Ka}}_{\mathrm{DBP}}{}^{**}\times \mathrm{DBP})}$$

$$*Ka_{\mathrm{alb}=}{}^{5.4\times }10^{4}M^{-1}$$

$$**{\mathrm{Ka}}_{\mathrm{DBP}}=3.7\;\mathrm{to}\;4.2\times 10^{7}\;M^{-1}$$

### Statistical analysis

Biochemical results are expressed as mean (SD) and p25-p75 (values for percentile 25 and 75 respectively) for each group. Comparisons between groups were carried out by t-student or Wilcoxon test for independent samples depending on the normality tests checks. For comparisons of more than three categories of a variable, Anova or Kruskall-Wallis test was used depending on the normality test checks (Shapiro-Wilks test) and homocedasticy test (Bartlett test). The correlation coefficient of Spearman was used along with its associated significance to verify the relationship between variables of continuous type. A value of p<0.05 was considered as indicative of significant differences. Statistical analysis was performed using the R Program (R Development Core Team), version 3.4.3. R: A language and environment for statistical computing. Vienna, Austria. (ISBN 3-900051-07-0).

## Results

A total of 241 children (60% males) were enrolled into the study. Five cases were excluded as a result of insufficient blood sample. Characteristics of the participants are shown in Table 1.

**Table 1:** Characteristics of the children.

Global n* = 236	n* (%)
**Sex**	
Boys	141 (59.7)
Girls	95 (40.3)
**Age Group**	
Neonates	47 (19.9)
0–2 years	33 (14)
2–6 years	79 (33.5)
>6 years	77 (32.6)
**Ethnicity** (n = 192)^	
Caucasian	170 (88.5)
Other	22 (11.4)
**Body Mass Index** (n = 214)^	
Obesity	12 (5.6)
Overweight	23 (10.7)
Normal weight	179 (83.6)

* n = sample size

^ This information could not be collected in 100% of the sample.

Free and total 25OHD, PTH and albumin concentrations differed significantly among all groups of age (p<0.001). Infants aged 0–2 years commonly recommended to take vitamin D prophylaxis in our community from birth to 12–18 months had higher concentrations of free and total 25OHD levels (p<0.001). However, newborns from mothers receiving nutritional supplements containing vitamin D during the gestation did not have significantly higher free 25OHD concentrations.

DPB concentrations were measured in 70 children and significant differences by age were found, being lower in the neonates’ group (p < 0.02) (Table 2). Sex and BMI did not influence on DBP concentrations (p = 0.69 and 0.53, respectively). Correlation between directly measured and calculated 25OHD was high and statistically significant (r = 0.66, p<0.001)

**Table 2:** Laboratory findings in serum of children grouped by age.

	Mean±SD	Median	Percentile 25	Percentile 75
Total 25OHD* **(ng/mL)**				
Newborns	19.99 ± 10.64	18.87	11.70	24.38
0–2 years	43.87 ± 12.32	43.77	37.20	51.56
2–6 years	28.89 ± 9.70	29.60	22.75	33.55
>6 years	23.03 ± 8.21	21.08	17.52	28.66
Free 25OHD* **(pg/mL)**				
Newborns	2.48 ± 1.39	2.26	1.68	2.95
0–2 years	5.46 ± 3.12	5.36	3.01	7.22
2–6 years	4.12 ± 1.72	4.28	2.92	5.44
>6 years	3.82 ± 1.43	3.54	2.78	4.94
PTH* **(pg/mL)**				
Newborns	20.30 ± 22.62	5.75	3.57	36.89
0–2 years	20.04 ± 10.32	15.26	12.44	27.27
2–6 years	25.44 ± 10.64	25.00	18.05	31.12
>6 years	31.94 ± 11.63	30.00	24.41	36.82
**DBP¶ (nmoL/mL)**				
Newborns	28.00 ± 3.90	28.25	26.57	30.01
0–2 years	35.77 ±7.93	33.47	31.42	39.49
2–6 years	37.95 ± 4.78	37.95	34.03	41.38
>6 years	35.57 ± 5.38	35.61	32.22	38.45
Albumin* **(g/dL)**				
Newborns	39.30 ± 3.50	40.10	37.40	41.20
0–2 years	43.44 ± 8.35	44.45	42.33	47.55
2–6 years	46.14 ± 2.33	46.60	44.98	47.90
>6 years	46.95 ± 2.85	46.60	45.20	48.40

**25OHD:** 25 hydroxyvitamin D. **PTH:** Parathyroid hormone. **DBP**: Vitamin D binding protein

*Significant differences detected among all groups of age (p Value <0 .001)

¶Significant differences detected among neonates group and the other groups of age (p Value < 0.05)

**Fig 1:**
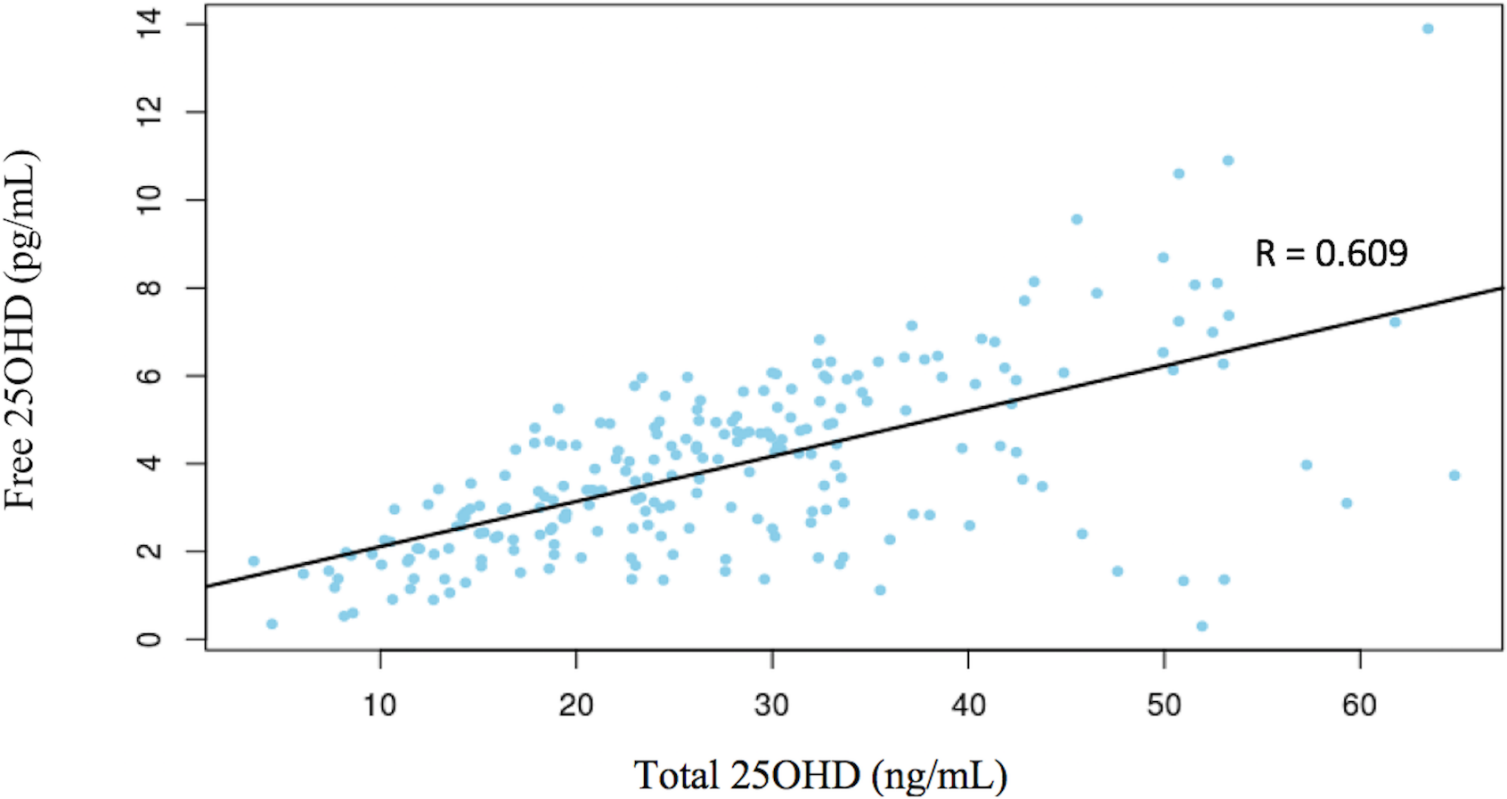
Significant (p < 0.001) positive correlation between serum free and total 25OHD concentrations.

For the whole sample, there was as well a high and significant correlation between serum free and total 25OHD levels (r = 0.61, p <0.001) (Fig 1).

**Fig 2:**
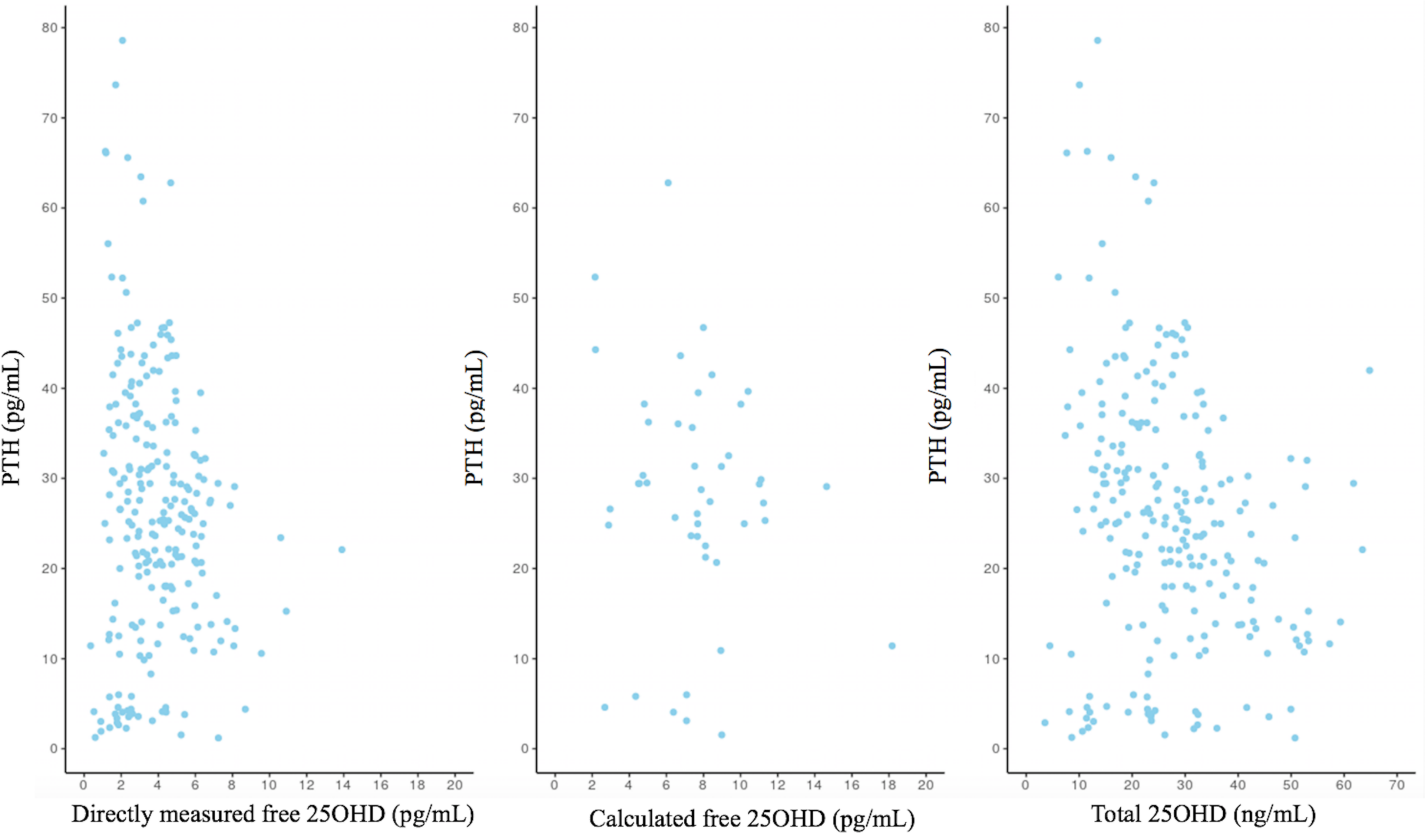
The correlation between the three different 25OHD values and PTH concentrations.

The correlation between measured and calculated serum free 25OHD and PTH concentrations was not significant, but it was significant between serum 25OHD total and PTH concentrations (Fig 2).

**Fig 3:**
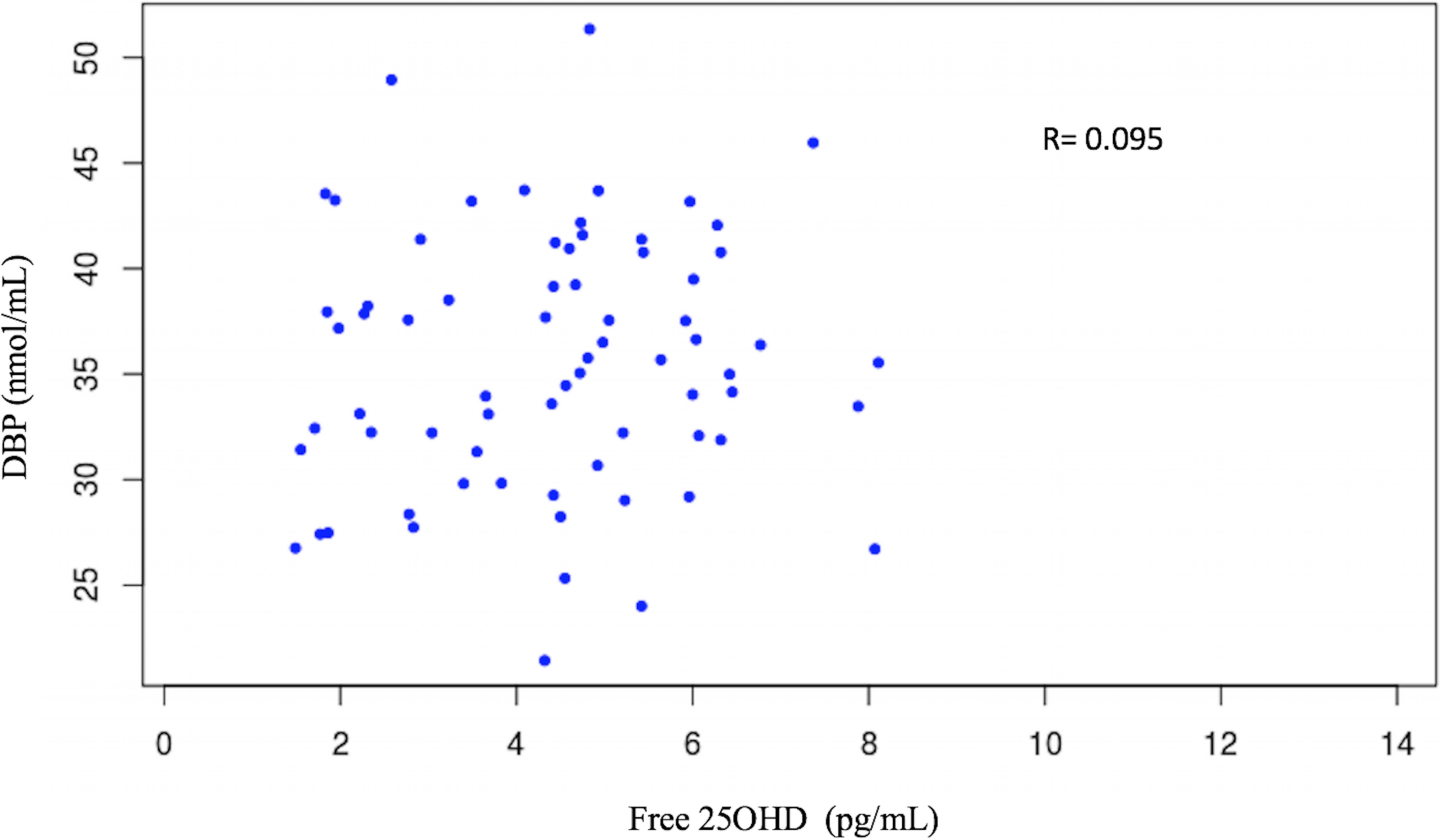
No significant correlation between serum free 25OHD and DBP concentrations.

**Fig 4:**
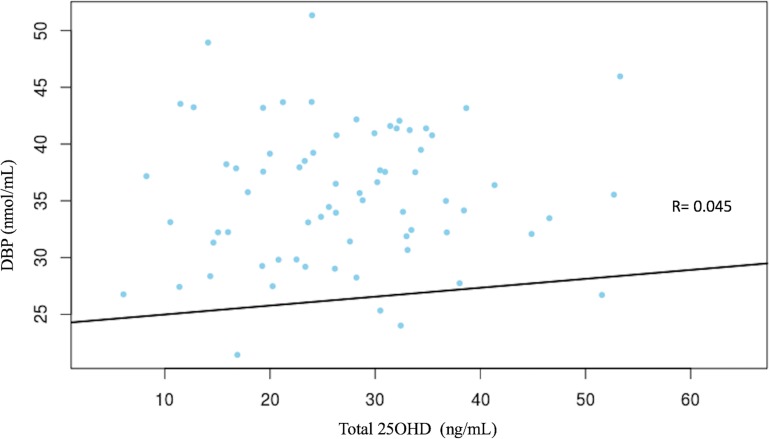
No significant correlation between serum total 25OHD and DBP concentrations.

Neither free nor total 25OHD concentrations were correlated with DBP levels (Figs 3 and 4).

The correlation coefficients between variables are shown in Table 3. Comparisons between groups demonstrated no effect of sex, ethnicity, BMI, diet, and sun exposure on free 25OHD levels (p>0.05). No differences were found between season of the year and free 25OHD values when the whole sample was analyzed globally (p = 0.26) However, the group of children older than 6 years had higher values (p = 0.003) in spring/summer than in autumn/winter. This significant elevation of free vitamin D did not correlate with a decrease in PTH.

**Table 3:** Correlations between variables in serum.

	Correlation	p Value
Free 25OHD*/Total 25 OHD	0.609	<0.001
Free 25OHD/PTH	-0.079	0.234
Free 25OHD/DBP	0.095	0.435
Free 25OHD/ Albumin	0.141	0.058
Total 25OHD/PTH	-0.253	<0.001
Total 25OHD/DBP	0.045	0.709
Total 25OHD/Albumin	0.116	0.115
Free 25OHD /Calculated free 25OHD	0.657	<0.001
Free 25OHD calculated/PTH	0.104	0.489

*Free 25OHD = measured free 25OHD

## Discussion

This study provides reference values of free serum 25OHD concentrations in a relatively large group of healthy children, mostly Caucasian, aged from birth to 14 years living in a region at northern latitude. To our knowledge, former data on values of free 25OHD in healthy paediatric population have not been published. Importantly, the study also shows a high correlation between free and total serum 25OHD concentrations. Infants aged 0–2 years had higher free and total 25OHD concentrations, likely resulting from oral vitamin D prophylaxis in this age’s group. The same result has been found in patients with cystic fibrosis that were receiving vitamin D supplements [22]. Thus, chronic supplementation with oral vitamin D increases free and total serum 25OHD concentrations in parallel [25].

The study also shows that serum free 25OHD and PTH concentrations are not significantly correlated whereas the relation between total 25OHD and PTH is inverse and significant, although weak. In 2005, Hollis et al. showed a clear inverse correlation between total 25OHD and PTH levels in adults, describing a clear plateau effect and a concentration from which on the decrease of 25OHD is associated with a rapid rise in PTH levels. This value, corresponding to a serum 25OHD level of 20 ng/ml, was subsequently defined as the deficiency threshold [26]. Although controversial, this value was also admitted in paediatric population [25]. However, studies carried out in infants and children did not confirm this cut-off point and it has not been demonstrated the appropriateness of this concentration, below 20 ng/ml, to define vitamin D deficiency in paediatric age [27,28]. Several organizations and societies have strived to developed guidelines for recommended desirable 25OHD levels, but their applicability across age is still debated. The American Society for Bone and Mineral Research proposes a desirable range of 25OHD concentrations of 20 to 40 ng/mL, whereas lower levels, 15 to 20 ng/mL, may be sufficient for infants and adults, and higher levels, 40 to 60 ng/mL, may still be safe [29]. Regardless of this limitation, as change of circulating PTH levels is considered to be one of the most sensitive biomarkers of vitamin D activity in vivo, the findings of our study indicate that the measurement of free vitamin D is not preferable to total 25OHD determination in the clinical estimate of vitamin D status in healthy children at any age.

Until few years ago, free 25OHD serum concentrations were usually calculated by means of a complex mathematical formula including total 25OHD and albumin values [4,11]. Recently, an assay to directly measure free vitamin D in serum samples has been developed and it is now available in the market (Future Diagnostics BV). Some studies have compared both methods of assessment, concluding that the direct measurement of free vitamin D is more accurate than that calculated [21,24]. The arising of the “free hormone” hypothesis [15] giving a predominant role to the unbound circulating 25OHD in the regulation of vitamin D bioactivity and the interest for the diagnosis of subclinical status of vitamin D deficiency, as mentioned above, might prompt the extended measurement of free 25OHD concentrations in serum, a technique with limitations such as the lack of a reference method and the low concentrations of free vitamin D detected [30]. It is of note that some children had high values of total but not free vitamin D (Fig 1) which can likely be explained by the cross reactivity with the 3-epi-25OHD.

Our results do not support that the determination of free 25OHD is more reliable that the measurement of total 25OHD as marker of vitamin D status in healthy paediatric population.

Relative to DBP levels, we found no significant relationship between free 25OHD and DBP concentrations. Powe et al. [14] reported that DBP concentrations were lower in African Americans than in whites and as a result, calculated bioavailable 25OHD concentrations derived from those DBP measures were similar in both populations concluding that low total 25OHD does not necessarily indicate low bioavailable form of vitamin D. However, other publications did not report differences in DBP, and issues have been raised concerning the DBP measurements used by Powe et al [31–33]. In addition to the absolute value of DBP concentrations, the DBP genotype might have different binding affinity for specific vitamin D metabolites and exert a significant effect on the concentrations of serum free 25OHD [34]. It has been suggested that the measurement of free 25OHD in serum may help to evaluate vitamin D status in protein deficient adults with liver insufficiency or malnutrition [8,16,17]. In these patients having low concentrations of carrier proteins (DBP or albumin), the free fraction of vitamin D was more strongly associated with mineral bone parameters than the total 25OHD. Our study, carried out in healthy children, did not address this issue. In preterm infants whose serum protein and albumin concentrations are usually low, free 25OHD concentrations did not significantly correlate with DBP levels and, as occurred in our study, highly correlated with total 25OHD concentrations [35].

Factors known to influence serum concentrations of total 25OHD, such as skin pigmentation, sun exposure, unbalanced dietary intake [36–39], were not significantly associated with free 25OHD concentrations in our study. Likewise, no association was found between BMI values and free 25OHD levels. No uniform results have been reported in publications analyzing the relationship between BMI and total 25OHD concentrations in paediatric population [40,41]. As expected and commented above, the sustained administration of vitamin D supplements increased the circulating concentrations of free and total 25OHD. This variable and the season of the year were the only environmental factors found to have a significant influence on the serum concentrations of free 25OHD in our sample of children including a large range of ages. Several studies report strong seasonal variation in 25OHD and PTH levels, and even a seasonal, inverse relationship between this parameter, recommending that the month of testing for vitamin D and PTH should be considered [42].

It should be kept in mind that the use of a questionnaire to get information on the other environmental variables is a methodological limitation of our study and attenuates the validness of the results. It is also of note that the sample of children was mostly composed of Caucasian individuals and the results cannot be directly extrapolated to populations having more racial variations.

## Conclusions

In summary, this study provides novel information on the values of free 25OHD serum concentrations in infants and children. The study indicates that, although the clinical application of free 25OHD measurement still needs to be established, the high correlation found between free and total 25OHD serum concentrations and the absence of significant correlation between free 25OHD and PTH levels suggest that the measurement of free 25OHD in serum does not represent any benefit on that of total 25OHD as marker of vitamin deficiency in healthy paediatric population.
